# Comparing the Effectiveness of a Web-Based Application With a Digital Live Seminar to Improve Safe Communication for Pregnant Women: 3-Group Partially Randomized Controlled Trial

**DOI:** 10.2196/44701

**Published:** 2023-07-24

**Authors:** Lukas Kötting, Christina Derksen, Franziska Maria Keller, Sonia Lippke

**Affiliations:** 1 Psychology & Methods Constructor University Bremen Germany

**Keywords:** Health Action Process Approach, HAPA, intention, safe communication behavior, patient safety, obstetric patients, digital intervention, web-based app

## Abstract

**Background:**

Medical internet interventions such as asynchronous apps and synchronous digital live seminars can be effective behavior change interventions. The research question of this study was whether digital interventions based on the Health Action Process Approach can improve pregnant women’s safe communication and patient safety in obstetric care.

**Objective:**

This study aims to compare a digital live seminar with a web-based application intervention and a passive control group and to identify which social cognitive variables determine safe communication behavior and patient safety.

**Methods:**

In total, 657 pregnant women were recruited, and hereof, 367 expectant mothers from 2 German university hospitals participated in the pre-post study (live seminar: n=142; web-based app: n=81; passive control group: n=144). All interventions targeted intention, planning, self-efficacy, and communication of personal preferences. The 2.5-hour midwife-assisted live seminar included exercises on empathy and clear communication. The fully automated web-based application consisted of 9 consecutive training lessons with the same content as that of the live seminar.

**Results:**

Controlled for sociodemographic characteristics, repeated measures analyses of covariance revealed that pregnant women significantly improved their self-reported communication behavior in all groups. The improvement was more pronounced after the digital live seminar than after the web-based application (*P*<.001; η_p_^2^=0.043). Perceived patient safety improved more for pregnant women participating in the live seminar than for those participating in the web-based application group (*P*=.03 η_p_^2^=0.015). A regression analysis revealed that social cognitive variables predicted safe communication behavior.

**Conclusions:**

Overall, the web-based application intervention appeared to be less effective than the digital live training in terms of communication behavior. Application interventions addressing communication behaviors might require more face-to-face elements. Improving intention, coping planning, and coping self-efficacy appeared to be key drivers in developing safe communication behavior in pregnant women. Future research should include social learning aspects and focus on the practical application of medical internet interventions when aiming to improve pregnant women’s communication and patient safety in obstetrics.

**Trial Registration:**

ClinicalTrials.gov NCT03855735; https://clinicaltrials.gov/ct2/show/NCT03855735

## Introduction

### Background

Medical internet interventions, such as asynchronous applications and synchronous digital live seminars, can be effective behavior change interventions [1]. In health care, several digital approaches have been used to improve patient safety [2-4]. Especially during the COVID-19 pandemic, digital interventions gained significance in reducing the risk of infection through personal contact [5]. Owing to lower costs, a potentially broader reach, and reduced logistic hurdles, research has focused on asynchronous training apps. Nevertheless, implementing an intervention can only be successful if the circumstances and stakeholder interests are considered [6].

One of the fields in which digital interventions could be especially useful is antenatal education and care [7]. Respectful maternity care, including safe and respectful communication, is an important aspect of obstetric practice and research [8]. Health care workers (HCWs) are encouraged to offer evidence-based care while also focusing on the personal needs and preferences of pregnant women and their families [8,9]. For a positive labor and birth experience, safe communication between the HCW and pregnant women and their families in a trusting, respectful, and empathetic environment is necessary [10]. In such an environment, pregnant women can openly share their feelings and needs [11-13].

Safe communication can be described as a multilateral process that involves sharing emotions, cognitions, and actions on a verbal and nonverbal level [14]. Although previous literature has mainly focused on communication competencies among HCWs, the literature is lacking in understanding safe communication behavior from the perspective of pregnant women and their families [15,16]. Previous research [9] showed that more than one-third of pregnant women reported that they felt unsure to ask questions or raise concerns while giving birth [17]. The reasons were perceived time constraints of HCWs, perceived power differences, and the worry of being perceived as a burden [17,18]. Despite these barriers, pregnant women’s communication competencies are rarely considered during antenatal digital interventions. As a result, the literature on digital safe communication trainings for pregnant women is currently scarce and lacks evidence.

This is particularly important because poor and ineffective communication in health care settings is a contributing and leading factor for adverse events that are a threat to patient safety, according to the report of the Joint Commission [19]. Patient safety is the absence of harmful events that could have been prevented under the given circumstances; for example, by safe communication [20]. Both preventable and nonpreventable adverse events may lead to detrimental outcomes for patients [21-23]. In obstetric care, not only the mother but also the unborn child might be affected by adverse treatment processes, such as inadequate patient-provider communication, which have the potential to result in preventable adverse events [24,25].

It is evident that effective communication is a prerequisite for safe care in obstetrics. Communication behavior can thus be seen as a crucial preventive health behavior [15]. There is extensive literature on health behavior change, indicating that multiple psychological and social factors have to be addressed. A variety of theories and models have been developed and applied to explain and predict behavior change [26]. However, many theories and models struggle to predict not only intention but also the translation of a behavioral intention (“I will always voice my concerns”) into behavior. There can be situational barriers, for example, pain and exhaustion during birth, as well as a lack of volitional factors such as coping planning [27]. A model that focuses on bridging this so-called intention-behavior gap to achieve behavior change is the Health Action Process Approach (HAPA) that examines social cognitive determinants of behavior [28].

The HAPA model assumes 2 distinct phases: first, in the motivational phase, an intention to act (in this case, to safely communicate with the HCW) is developed based on the individual’s outcome expectancies and risk perceptions. In the second phase, the volitional phase, this intention is brought into action through planning. During all the stages of behavior change, situational barriers and facilitators intercorrelate with this process [28]. Self-efficacy beliefs are crucial for planning, adopting, and maintaining a new behavior [29].

To actually improve safe communication behavior instead of the intention to communicate safely and thus reduce potential preventable adverse events, digital interventions must be tailored to the social cognitive barriers and facilitators for pregnant women. Previous literature has demonstrated that interventions based on motivational and volitional theories, such as the HAPA, are effective in improving health-related behaviors such as safe communication [16,30,31]. The HAPA model has rarely been applied to predict and improve safe communication behaviors in health care [16,31]. Most interventions are solely offered to the HCW.

### Current Research

Taken together, pregnant women’s safe communication behavior in the context of obstetrics requires further examination, especially regarding digital antenatal communication interventions during the COVID-19 pandemic. Therefore, this study will evaluate 2 digital interventions that are hypothesized to improve perceived communication behavior and perceived patient safety within the sample of pregnant women and investigate behavior change determinants. The aim of this study was to compare the effectiveness of a web-based application intervention with a digital live seminar and a passive control group (CG).

Thus, the hypotheses are as follows:

- Hypothesis 1: pregnant women who use the web-based application before giving birth will show greater improvement in the primary outcome of safe communication behavior and the secondary outcome of perceived patient safety than women from a passive CG. Their improvement was comparable with an intervention group that received a web intervention (web live seminar).
- Hypothesis 2: the HAPA model can explain the safe communication behavior of pregnant women after web-based application interventions; coping planning, coping self-efficacy, and intention are associated with safe communication behavior after digital interventions.
- Hypothesis 3: perceived patient safety is associated with safe communication behavior after web-based application use.

## Methods

### TeamBaby Project

Data collection took place within the TeamBaby Project, which aimed to investigate and improve the psychological mechanisms underlying safe communication behavior in obstetrics, specifically before and during birth. The TeamBaby Project is funded by the German Innovation Fund (project number 01VSF18023) of the Gemeinsamer Bundesausschuss (G-BA) and registered with ClinicalTrials.gov (ClinicalTrials.gov identifier: NCT03855735).

### Recruitment and Procedures

#### Participants

All participants were pregnant women intending to give birth at 1 of 2 project-affiliated university hospitals providing the highest level of care in affiliated neonatal intensive care units. Within the 2 university hospitals, expecting mothers were approached by a project-affiliated study nurse and a research associate. Recruitment was facilitated by distributing flyers, posters, and registration forms at key locations (antenatal clinics, waiting rooms, wards, corridors, and lifts) as well as through social media posts. In addition, gynecologists in private practice, midwives, counseling centers, and pharmacies were approached with additional material to distribute to their clients. Participants registered via email by filling in a registration form. During the registration process, participants were provided with an informed consent form offering a detailed description, including the outcomes of the study. Furthermore, as part of the informed consent, participants were informed of whether they were randomly allocated to the intervention or passive CG. In addition, participants were informed that no harm or unintended effects were expected as part of their participation. To ensure the privacy and confidentiality of the obtained data, each pregnant woman was asked to generate a unique pseudonymization code and subsequently received a baseline questionnaire (provided that informed consent was given) afterward. Further inclusion criteria were sufficient knowledge of German and age of maturity (>18 years). Expectant mothers created their own participant codes, using the following scheme: (1) the first 2 letters of the father’s surname, (2) the first 2 letters of the mother’s surname, and (3) the birthday of the expectant mother. In accordance with the data security approval obtained, the process of pseudonymizing the data allowed for no conclusions regarding personal data.

Between June 2020 and August 2021, participants were randomized to either an intervention group that received a digital live seminar training (live seminar group [IG1]) or a passive CG. Women in the passive CG were fully informed about the study, including the possible intervention, before the randomization. They did not receive any additional intervention or educational material. The randomization for the live seminar was prepared and performed by project-affiliated team members (study nurses and research assistants) at the 2 hospitals. For this purpose, closed envelopes were prepared in a ratio of 3:2 for 77.6% (222/367) of pregnant women.

Although a complete randomization was planned, 52 (36.1%) of 144 women had to be allocated to the CG owing to their expected due date. To ensure that the live seminar worked in a group setting, 12 (8.3%) of 144 women who provided postpartum survey data for the intervention group were not randomized but were assigned when the live intervention started between August 2021 and June 2022, and a third group of expectant mothers were recruited for the web-based application intervention (web-based application group [IG2]). Participants were informed of the study in writing and asked to provide informed consent before participation. As with the digital live trainings, participants were asked to create a unique pseudonymization code to ensure that all privacy and confidentiality regulations were met as part of the data collection and evaluation.

Different recruitment periods were planned from the beginning to avoid overlapping recruitment efforts at the 2 hospitals. Nevertheless, the recruitment of pregnant women for the digital live seminar took place during COVID-19 pandemic restrictions, including access restrictions for both support persons and the study personnel. Participants answered the survey questions twice (before and after birth), with the abovementioned interventions conducted before giving birth. The participants did not receive any form of compensation for their participation in the study.

**Figure 1:**
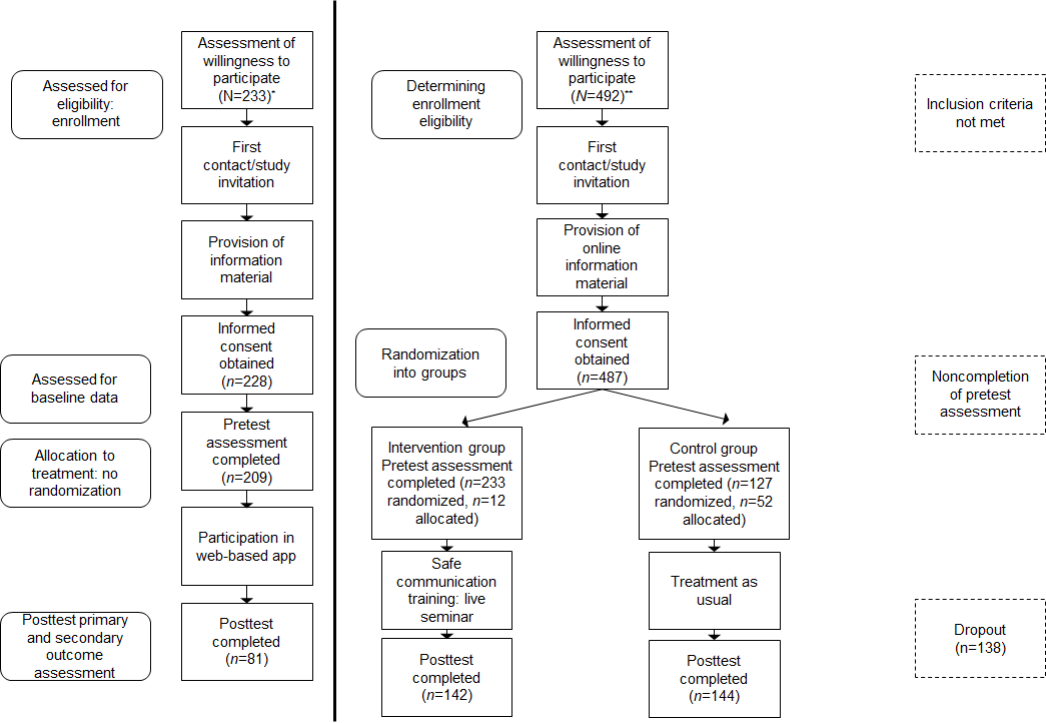
Flowchart of study participation. *Study flow for the web-based application intervention group. **Previous live seminar intervention and the passive control group.

A detailed description of the recruitment process and dropout from the interventions is shown in Figure 1.

#### Interventions

For the digital live seminar, content for communication training was developed by 2 organizations that consulted on patient safety and communication in collaboration with the research team. The content was delivered through a 2.5-hour web session facilitated by 2 communication trainers, including a *physician* and midwife. Details of the training provided to the pregnant women and their partners have been described elsewhere [32]. The HAPA and Behaviour Change Taxonomy were used to guide intervention development [33]. To prepare for the live seminar, pregnant women completed a self-reflection questionnaire regarding their birth preferences. The live seminar consisted of an introduction round to uncover individual needs and potential communication approaches. Subsequently, an exercise on perspective taking (“empathy maps”) was included to invite participants to take the point of view of the HCW. Then, the pregnant women practiced communication competencies while considering typical situations in obstetrics. “Speaking up” to voice own concerns and “closed-loop communication” to facilitate mutual understanding were introduced. Finally, participants were invited to develop a behavioral plan regarding the communication of their individual needs.

The training used in the web sessions was adapted for the fully automated web-based application intervention. The web-based application was also based on the HAPA and behavior change techniques (BCTs) [33], aiming to (1) raise awareness of the importance of communication behavior, (2) create an intention to apply communication strategies, and (3) raise belief in one’s ability to use and implement strategies. In line with the live seminar targeting pregnant women’s safe communication behavior, the psychological interventions implemented in the web-based application focused on BCTs that could be linked to the HAPA. These included goal setting (outcome; BCT 1.3), commitment (BCT 1.9), monitoring of emotional consequences (BCT 5.4), instruction on how to perform the behavior (BCT 4.1), discrepancy between current behavior and goal (BCT 1.6), information about health consequences (BCT 5.1), and feedback and monitoring (BCT 2).

Content and adaptations for the German web-based application were developed with physicians from 2 university hospitals (n=4), psychologists (n=4), and the German Alliance for Patient Safety. The content from the web training was further iterated by project researchers (psychologists), obstetricians at clinics, and web application developers. The development process included a beta version of the web-based application tested by an affiliated health insurance company. The web-based application was accessible through all web browsers. It consisted of 10 consecutive lessons, from basic communication competencies to action plans. The details of the modules in the web-based application are provided in Tables S1 and S2 in Multimedia Appendix 1.

#### Measures

##### Overview

The primary outcome of the study was pregnant women’s communication behavior, and the secondary outcome was perceived patient safety. As behavioral determinants, action planning and coping planning were assessed using self-reported questionnaires. Items stemmed from previously validated scales [34-36], which were revised by the project team (obstetricians and health psychologists). The questions were administered in German.

##### Communication Behavior

Communication behavior was assessed via 7 items from a self-constructed scale based on the communication competencies by Rider and Keefer [35], “During pregnancy, I have communicated my needs clearly.” The answer categories ranged from 1 (does not apply at all) to 6 (applies fully and completely), with a Cronbach α of .63 at the first time point (T1) and .81 at the second time point (T2).

##### Perceived Patient Safety

Perceived patient safety was measured as perceived patient safety risks with 9 items that were adapted to the pregnant women’s perspective from a self-constructed and previously validated scale [37], “Before, during and after birth, I observed at least once that not enough healthcare workers were present.” The answer categories ranged from 1 (does not apply at all) to 6 (applies fully and completely) at baseline and 1 to 4 in the questionnaire after birth, with a Cronbach α of .82 at T1 and .85 at T2. Baseline values were recorded using the formula “Y = (B − A)*(x − a)/(b−a) + A,” with the old minimum (a), new minimum (A), old maximum (b), and new maximum (B) [38]. Higher levels indicate more perceived risks and thus a lower perceived safety.

##### Coping Planning

Coping planning was measured with a single item based on previously validated items in other behavioral domains [34]: “I was able to practically apply my plans for communicating during birth, even when encountering difficulties.” The answer categories ranged from 1 (*much lower compared with other patients*) to 5 (*much higher compared with other patients*).

##### Coping Self-Efficacy

Coping self-efficacy was assessed via a self-constructed single item on the basis of previously validated items in other behavioral domains [34]: “I was sure I could communicate well even when I was tired or exhausted.” The answer categories ranged from 1 (does not apply at all) to 6 (applies fully and completely).

##### Intention

Intention was assessed via 2 self-constructed items on the basis of previously validated items in other behavioral domains [34], “I intend to always pay attention that I communicate safely with the doctors and midwives.” The answer categories ranged from 1 (does not apply at all) to 6 (applies fully and completely), with a Spearman ρ of 0.71 at T1.

#### Sociodemographic Data

Age, marital status, highest level of education, and nationality were assessed in categorical data. Age (1: “younger than 20 years of age”; 2: “20-29 years”; 3:“30-39 years”; 4: “40-49 years”), education (1: “middle school degree or lower”; 2: “high school diploma”; 3: “vocational training”; 4: “university degree”), and marital status (1: “single”; 2: “in a relationship”; 3: “married”; 4: “divorced/separated”) were measured in 4 categories. Nationality (1: “German” or 2: “Other”) was measured dichotomously.

#### Statistical Analysis

All analyses were conducted using SPSS software (version 29.0; IBM Corp). The authors were not blinded to the analysis. Regarding hypothesis 1, 2 repeated measures analyses of covariance were used to examine and compare changes in safe communication behavior and patient safety across the 3 groups (IG1, IG2, and CG) in a pre-post design. For the repeated measures analyses of covariance, age, nationality, relationship status, and education were recoded into binary variables, so they could be added as covariates. For age, 2 binary variables were used to compare younger patients with patients in the age range of 30 to 39 years and older patients with patients in the age range of 30 to 39 years. Nationality was recoded to compare German participants with pregnant women of a different nationality. Relationship status was recoded to compare pregnant women currently in a relationship with those currently single for different reasons (including separated or divorced). The educational level was recoded as “university degree” versus “other.” Finally, the group factor was added as 2 binary variables to compare IG2 with IG1 and the CG. To test the drivers of safe communication behavior (hypothesis 2) and the association of patient safety with communication behavior (hypothesis 3), regression paths based on the HAPA model were analyzed for all 3 groups (IG1, IG2, and CG). Table S3 in Multimedia Appendix 1 shows the partial intercorrelations between variables. In the partial correlations of the studied variables, age, marital status, education level, and nationality were included as control variables. Missing values occurred in ≤5% of all cases. Thus, missing data were handled via listwise deletion.

### Ethics Approval

The Declaration of Helsinki was adequately addressed, and this study was approved by the Ethics Committee for Human Research of the University Hospital Ulm (number 114/19) and the Ethics Committee for Medical Research of the University Hospital Frankfurt (number 19-292). Approval for this study was obtained without any exemption.

## Results

### Participants

In total, 367 (IG1: n=142; CG: n=144; IG2: n=81) expectant women participated in the 2 survey waves, while providing matchable codes, and were thus included for data analysis. Figure 1 depicts the details of the participation process and dropouts, including all expectant mothers who had originally intended to participate in the study (IG1: n=225; CG: n=199; IG2: n=233). Dropout between the 2 survey waves (IG1: n=83; CG: n=55; IG2: n=152) occurred in the following cases: delivery at another clinic, no completion of the second survey wave, preterm delivery before the web intervention or web-based application, and delivery-related health complications. As highlighted in Figure 1, there were cases in which participants could not be randomized and were thus allocated to either the control or intervention group because of upcoming delivery dates.

Table 1 provides an overview of the sociodemographic data. Most participants were aged between 30 and 39 years, predominantly well educated (a university degree), married or in a stable partnership, and of German nationality.

**Table 1:** Sociodemographic characteristics and intervention group affiliations of expectant mothers.

Items		IG1^a^ (n=142), n (%)	CG^b^ (n=144), n (%)	IG2^c^ (n=81), n (%)	Missing values^d^, n					
					IG^e^		CG		App	
**Age group (years)**										
	>20	N/A^f^	N/A	N/A	N/A		N/A		N/A	
	20-29	14 (9.9)	19 (13.2)	9 (11.1)	0		4		5	
	30-39	119 (83.8)	107 (74.3)	57 (70.4)	N/A		N/A		N/A	
	40-49	9 (6.3)	14 (9.7)	10 (12.3)	N/A		N/A		N/A	
**Marital status**						0		4		5
	Single	2 (1.4)	3 (2.1)	3 (3.7)						
	In a committed relationship	34 (23.9)	27 (18.8)	13 (16)						
	Married or registered partnership	106 (74.6)	109 (75.7)	60 (74.1)						
	Divorced or separated	N/A	1 (0.7)	N/A						
**Highest educational level**						0		4		5
	No school-leaving qualification	1 (0.7)	N/A	N/A						
	Secondary or elementary school leaving	N/A	N/A	1 (1.2)						
	Secondary school diploma	2 (1.4)	3 (2.1)	2 (2.5)						
	A levels	7 (4.9)	6 (4.2)	4 (4.9)						
	Completed vocational training	19 (13.4)	27 (18.8)	11 (13.6)						
	University degree^g^	27 (19)	26 (18.1)	17 (21)						
	University degree	86 (60.6)	78 (54.2)	41 (50.6)						
**Nationality**						0		4		5
	German	122 (85.9)	122 (84.7)	72 (88.9)						
	Other	20 (14.1)	18 (12.5)	4 (4.9)						

^a^IG1: live seminar group.

^b^CG: control group.

^c^IG2: web-based application group.

^d^Missing values for each group.

^e^IG: intervention group.

^f^N/A: not applicable.

^g^Special German university degree (Hochschule).

### Descriptive Statistics

Descriptive statistics on age, relationship, and education level, as well as the nationality of expectant mothers included in the study, are shown in Table 1. All descriptive descriptions of expectant mothers are subdivided with regard to the form of intervention and missing values, whereby the respective frequency and percentage are provided in Table 1.

In addition to Table 1, groups of participants were compared using chi-square tests for categorical sociodemographic data. The results indicated no differences between the groups in age (*χ*²_4_=4.2; *P*=.38, education level (*χ*²_12_=8.3; *P*=.76), marital status (*χ*²_6_=4.5; *P*=.61), or nationality (*χ*²_4_=5.5; *P*=.24). More detailed results are presented in Table S4 in Multimedia Appendix 1. Finally, participants who dropped out were compared with participants who provided T2 data in their respective study group using chi-square tests for categorical sociodemographic characteristics to test differences in the mentioned study groups and their sociodemographic characteristics (Table 1). Pregnant women who discontinued the web-based application differed from those who completed it and provided T2 data regarding their sociodemographic characteristics in terms of age with *χ*^2^_3_=49.7; *P*≤.001 and nationality with *χ*^2^_1_=5.9; *P*=.02. No significant differences were found in marital status (*χ*^2^_2_=2.3; *P*=.32) and education level (*χ*^2^_5_=3.2; *P*=.66). The same picture emerged for IG1 for age (*χ*^2^_3_=2.9; *P*=.39), nationality (*χ*^2^_1_=0.2; *P*=.67), marital status (*χ*^2^_2_=1.4; *P*=.49), and education level (*χ*^2^_6_=5.4; *P*=.50). Finally, no significant differences were found in the CG for age (*χ*^2^_2_=3.5; *P*=.17), nationality (*χ*^2^_2_=2.2; *P*=.34), marital status (*χ*^2^_3_=1.5; *P*=.68), and education level (*χ*^2^_5_=7.9; *P*=.16). The results of all the mentioned groups are depicted in Table S5 in Multimedia Appendix 1.

### Communication Behavior

**Figure 2:**
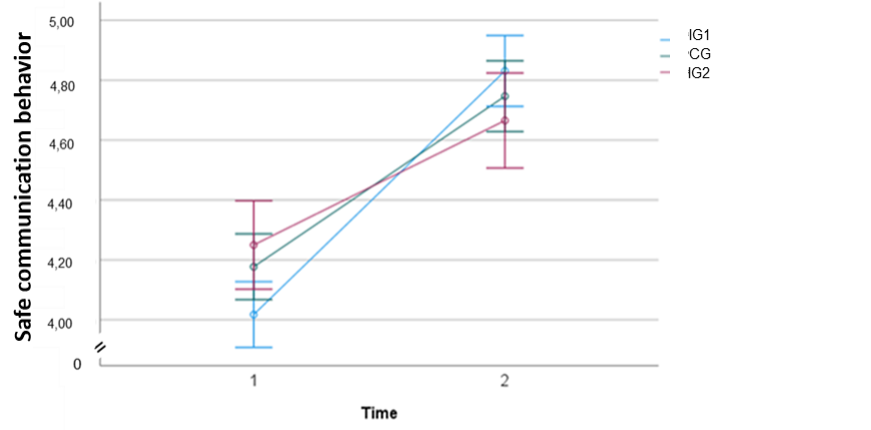
Estimated marginal means of safe communication behavior over 2 time points. CG: control group; IG1: live seminar group; IG2: web-based application group.

Regarding hypothesis 1, the main effect of time on communication behavior scores was not statistically significant (*F*_1,336_=3.322; *P*=.07; η_p_^2^=0.010). This suggests that across groups, the mean level of communication behavior scores did not exhibit a significant trend over the measurement points (Figure 2).

There was a significant time × group interaction effect, meaning that the change in communication behavior across time differed between IG1 and IG2 (*F*_1,336_=15.046; *P*<.001; η_p_^2^=0.043). Between the CG and IG2, no significant time × group interaction effect emerged (*F*_1,336_=2.732; *P*=.10; η_p_^2^=0.008).

### Perceived Patient Safety

**Figure 3:**
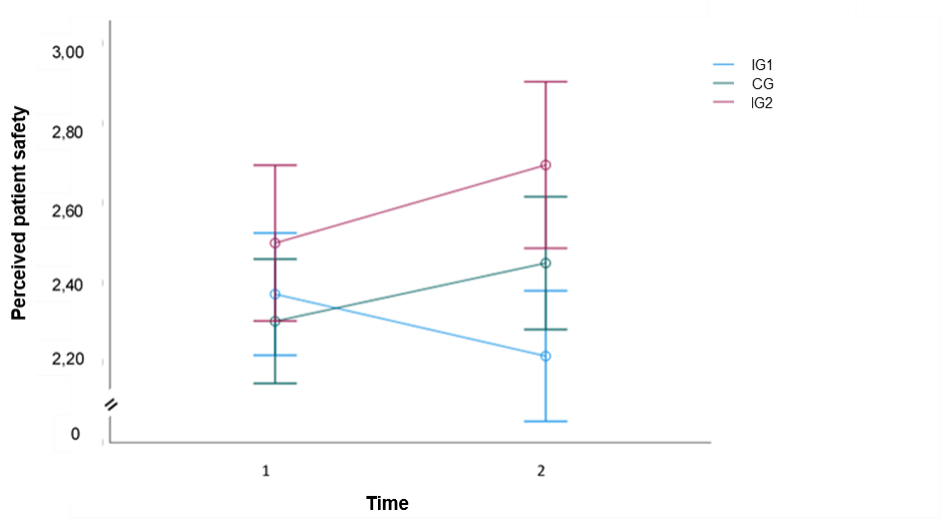
Estimated marginal means over 2 time points of perceived patient safety. CG: control group; IG1: live seminar group; IG2: web-based application group.

The main effect of time on perceived patient safety scores was not statistically significant (*F*_1,304_=0.013; *P*=.91). This suggests that, across groups, the mean level of patient safety scores did not exhibit a significant trend across the measurement occasions (Figure 3).

There was a significant time × group interaction effect, meaning that the change in perceived patient safety across time did significantly differ between IG1 and IG2 (*F*_1,304_=4.709; *P*=.03; η_p_^2^=0.015). There was no significant time × group interaction effect between CG and IG2 (*F*_1,304_=0.108; *P*=.74; η_p_^2^≤0.001).

**Figure 4:**
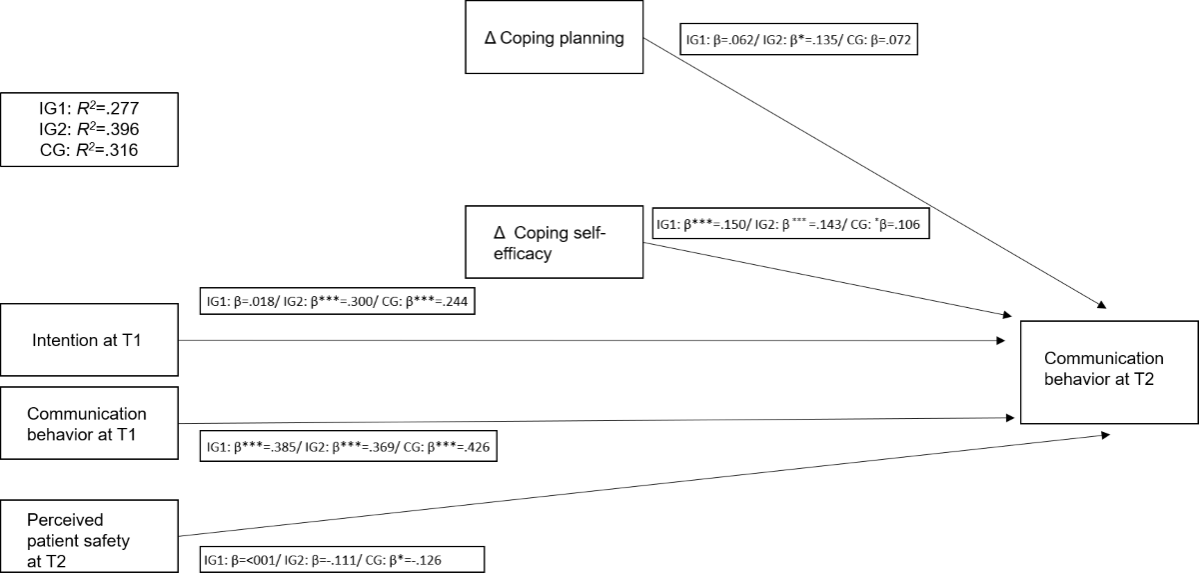
Regression model of social cognitive Health Action Process Approach variables and safe communication behavior across all groups. CG: control group; IG1: live seminar group; IG2: web-based application group. *β is significant at the *P*=.05 level. ***β is significant at the *P*=.001 level.

To investigate hypothesis 2 and assess whether social cognitive HAPA variables were associated with safe communication behavior after web-based application use, a multiple regression analysis was performed (Figure 4; Table 2).

**Table 2:** Results from the social cognitive regression model in the Health Action Process Approach framework across all 3 groups.

			B^a^ (95% CI; SE)		β^b^		*P* value		Tolerance		VIF^c^
**Parameters (web-based application group)^d^**											
	Intention T1^e^	0.300 (0.124 to 0.476; 0.088)		.351		.001^f^		0.794		1.260	
	Δ Coping self-efficacy	0.143 (0.015 to 0.272; 0.064)		.259		.03^g^		0.627		1.594	
	Δ Coping planning	0.135 (0.015 to 0.262; 0.064)		.270		.04^g^		0.522		1.915	
	Communication at T1	0.369 (0.007 to 0.598; 0.115)		.350		.002^h^		0.714		1.401	
	Perceived patient safety	−0.111 (0.140 to 0.025; 0.068)		−.153		.11		0.965		1.036	
**Parameters (live seminar group)^i^**											
	Intention at T1	0.018 (0.070 to 0.440; 0.088)		.018		.84		0.924		1.082	
	Δ Coping self-efficacy	0.150 (0.004 to 0.207; 0.044)		.290		.001^f^		0.672		1.488	
	Δ Coping planning	0.062 (−0.013 to 0.157; 0.038)		.142		.16		0.687		1.457	
	Communication at T1	0.385 (0.236 to 0.715; 0.088)		.369		.001^f^		0.916		1.092	
	Perceived patient safety	<0.001 (−0.250 to −0.002; 0.063)		<.001		>.99		0.881		1.135	
**Parameters (control group)^j^**											
	Intention at T1	0.255 (0.070 to 0.440; 0.093)		.221		.007^h^		0.924		1.082	
	Δ Coping self-efficacy	0.106 (0.004 to 0.207; 0.051)		.196		.04^g^		0.672		1.488	
	Δ Coping planning	0.072 (−0.013 to 0.157; 0.043)		.157		.10		0.687		1.457	
	Communication at T1	0.426 (0.236 to 0.615; 0.096)		.362		.001^f^		0.916		1.092	
	Perceived patient safety	−0.126 (−0.250 to 0.002; 0.063)		−.166		.047^g^		0.881		1.135	

^a^Unstandardized coefficient.

^b^Standardized coefficient.

^c^VIF: variance inflation factor.

^d^n=77.

^e^T1: first time point.

^f^B is significant at the *P*=.001 level.

^g^B is significant at the *P*=.05 level.

^h^B is significant at the *P*=.01 level.

^i^n=125.

^j^n=119.

As summarized in Table 2, safe communication behavior after web-based application use was significantly predicted by the reported intention to communicate safely at baseline. Safe communication behavior at baseline significantly predicted safe communication behavior after participation in the web-based application. Safe communication behavior was also significantly determined by a change in coping planning and a change in coping self-efficacy between time points.

Nevertheless, safe communication behavior after participating in IG1 was not significantly predicted by the reported intention to communicate safely at baseline (Table 2). Safe communication behavior at baseline significantly predicted safe communication behavior after participating in the live seminar. Behavior was also significantly determined by a change in coping self-efficacy but not in coping planning between time points.

Expectant mothers’ safe communication behavior in the CG was significantly predicted by their reported intention to communicate safely at baseline. Safe communication behavior at baseline significantly predicted safe communication behavior after giving birth. Finally, safe communication behavior was significantly determined by a change in coping self-efficacy but not by a change in coping planning between time points. All results across the abovementioned groups are presented in Table 2.

Regarding hypothesis 3, it could not be empirically supported that patient safety also played a role in communication behavior after using the web-based application; no significant association with perceived patient safety at the end of the observation period emerged with safe communication behavior. Similar results were observed for IG1. In the CG, an association emerged between perceived patient safety after birth and safe communication behavior. Notably, on a bivariate level, there were significant correlations between perceived patient safety and communication at T2 only in the CG (Table S1 in Multimedia Appendix 1).

## Discussion

### Principal Findings

This study aimed to compare and identify the effectiveness of different digital intervention modes for pregnant women regarding the primary outcome of safe communication behavior and the secondary outcome of perceived patient safety in obstetric care. It was hypothesized that participation in a digital web-based application would prove to be equally effective as a live seminar and more effective than a passive CG. However, this could not be empirically supported: compared with the intervention group, the pregnant women who used the web-based training application improved their safe communication behavior significantly less and not more than a passive CG that did not receive any intervention.

There are very few studies [39-41] on the effectiveness of (digital) interventions in the context of pregnant women’s safe communication behavior in obstetrics. Thus, hypotheses could only be drawn based on other literature concerning the HAPA framework [42-44]. Thus, it is even more important to gain evidence in this area of research, especially regarding behavior change interventions [42,45,46]. It seems that theoretical foundations regarding BCTs in communication research are lacking and that tangible BCTs are missing or insufficient, as the literature demonstrates [47].

There are several theoretical explanations for the lower effectiveness of the web-based application intervention. Expectant mothers using the web-based application might have started with a different general understanding of their own ability to communicate safely and also of what safe communication behavior entails. As Figure 2 shows, their baseline score is, on average, higher than that of the other 2 groups. In addition, the web-based application was a more rigid knowledge-based approach to teaching safe communication behavior as compared with the web seminar with its practical and interactive elements. However, the interactive element, even if only “on-screen,” might be crucial so that participants understand their own limits regarding safe communication and how to translate theoretical knowledge concerning communication behavior into action to bridge the intention-behavior gap. This is consistent with the finding of the dropout analyses that pregnant women with a different nationality than German were more likely to not complete the web-based application, probably because it was too text based. In contrast, there were no differences in the sociodemographic characteristics of women who completed the study and those who dropped out in IG1 and CG. The web-based application encompasses a knowledge-based learning experience [48] but no practical rehearsal in a natural environment, thus hindering potential learning and transfer effects. Although knowledge-based interventions can be effective in enhancing health literacy [49], health behavior change might only be possible if interventions are enriched with elements that target behavioral planning and enhancing self-efficacy [50].

In this context, professional and personal support can be perceived as trustworthy and knowledgeable [48,51]. In our digital live seminar, the trainers were experts in the field, thus providing guidance beyond the scope of the web-based application intervention. Furthermore, BCTs were not as effective in their implementation in the application intervention as found previously [32]. The implementation of BCTs in the application may not have worked as well as in the training, as BCT in the web-based application focused on the motivational phase of HAPA. However, more proximal factors, such as volitional factors, have been found to have a more direct and thus larger effect on behavior [52]. Compared with the live seminar, the web-based application offered fewer opportunities for individual action planning. Future research should evaluate which BCT is best for implementing volitional factors in digital interventions.

In previous literature, legitimacy has been identified as a crucial factor [51]. Digital and especially asynchronous tools are limited with regard to such effects, and notably, such elements were absent in the currently applied web-based applications. Digital interventions based on BCTs are already widely used for health maintenance, including the prevention and management of health problems [53]. Nevertheless, they might need to be revised under these considerations to provide the opportunity for contact with a trainer [48].

Contact with a trainer could also positively impact the user experience of pregnant women and their partners [54]. User barriers include the perception of irrelevant or unsuitable content, lack of time, and not having the option to save the digital tool on a mobile phone [54]. Consistent with the literature [55], there are 2 key characteristics of successful digital interventions on which the web-based application is improvable: inclusion of the target group in the development of the web-based intervention and the application of clear guiding principles. Guiding principles should be identified that answer key context-specific behavioral issues in the respective research field [55], such as a lack of respectful maternity care and patient involvement. Applied to the context at hand, context-specific stakeholders, including expectant mothers, their support persons, and HCW should be trained, and their communication strategies should be aligned. It should be noted that such elements have been included in developing the current version of the web-based application (eg, through tasks on perspective taking), although potentially more iterations could have been needed to adapt the web-based application even further to context-specific behavioral needs and preferences. Improving the web-based application on these points could lead to higher overall effectiveness, more closely resembling the effectiveness of a face-to-face intervention.

Consequently, it is necessary to identify effective mechanisms of (digital) interventions in addition to simply demonstrating their overall effectiveness [56]. We investigated the potential mechanisms in hypothesis 2 to understand what drives individual differences among participants regarding their improvements in safe communication behavior over time. For pregnant women who participated in the web-based application, coping planning and self-efficacy determined safe communication behavior. Various intervention studies have shown that both self-efficacy and coping planning can be trained in interventions targeting knowledge and self-regulation [42,57-59]. This indicates that a theoretical understanding and appraisal of safe communication behavior are important determinants of improvement among participants using the web-based application.

Notably, a different picture emerged in the live seminar and in the CG, where coping self-efficacy was the main determinant of pregnant women’s safe communication behavior. Thus, the web-based application stands out in the sense that multiple HAPA constructs predicted safe communication behavior at T2. Not only does the belief that one can communicate safely in difficult situations (coping self-efficacy) seem to play a role but also does the transfer of knowledge regarding concrete plans for these situations (coping planning). This further illustrates the need to incorporate elements regarding the social aspect of learning as well as further chances to translate theoretical knowledge into practice within a natural setting [60]. Ultimately, personalized or interactive elements seem to be an essential aspect in a variety of (digital) intervention studies [61]. One possibility would be to enrich the web-based application with a face-to-face format or a chatbot [62] to increase effectiveness and maintain practical advantages of digital training compared with a more extensive stand-alone, face-to-face intervention.

Another topic of concern was to investigate whether and how participation in the web-based application related to perceived patient safety. We expected that recipients of the web-based application would improve more than a passive CG and to a similar degree as recipients of a live seminar intervention. This was not empirically supported. In addition, an association between perceived patient safety and safe communication behavior emerged only in the CG. It is possible that participants in the CG had worse birth experiences and thus perceived higher patient safety risks. As safe communication behavior is central to good birth experiences, their perceptions of birth might have acted as a confounding factor in both perceived risks and communication. IG2 and IG1 focused on safe communication behavior, which is, although important, only one aspect of perceived patient safety and might be overshadowed by more obvious medical aspects and behavioral variables in this specific context, such as the birth experience. In this case, the web-based application in its current form was not able to improve perceived patient safety.

### Limitations and Recommendations for Future Research

This study was the first approach to design and apply a digital training tool in the form of a web-based application to improve expectant mothers’ safe communication. This study has several limitations. First, the lack of available previously published evidence negatively affected the ability to design and tailor such a web-based application to expectant mothers’ needs and the accuracy of the hypothesized effectiveness. Future research in this area will benefit from the insights generated in this study. Consecutive research designs should permit more rigorous testing and a thorough development phase for the design and content according to the needs of participants before using a medical internet intervention. Similarly, optimizing the intervention effect and user experience could be achieved by incorporating face-to-face elements or the possibility of social exchange into the digital intervention design [48].

From a methodological perspective, the group of web-based application users was smaller than the other 2 groups. Absolute sample size and potential distortions (eg, due to dropout issues and social desirability) might explain the difference between hypothesized and empirically observed group differences [63,64]. The use of the web-based application by expectant mothers took place without further observation or consultation with the research staff, which is why interference effects (eg, frequent interruption of an exercise, multitasking, or an environment) could not be controlled. The assessments were self-reported measures and thus potentially biased by subjective beliefs and social desirability. In addition, we used only subjective reports and single-item scales for reasons of feasibility, but they might have had low reliability. Therefore, future research might benefit from developing and further validating multi-item scales to assess safe communication behavior or using observation assessments for a more objective assessment. This could offer additional insights regarding potential subjectivity within self-reported measures.

In this study, mostly well-educated women participated, which probably had an effect on the results, and thus limited generalizability. The web-based application should also be tested and verified with other sociodemographic groups with lower levels of education and migration backgrounds [40,65]. Consequently, future studies should aim for a more diverse participant pool [40,66]. Collaboration with cultural associations or municipal services could aid in this strategy and the sustainability of the intervention [55].

In addition, it should be mentioned that the data collection in the live seminar between June 2020 and August 2021, the COVID-19 pandemic was associated with restrictions at the hospitals. For example, expectant fathers were partially not allowed to be present during the birth, and interpersonal contacts were limited to a minimum to prevent the spread of the pandemic. All these points may have had an impact on the communication within the hospitals, for example, due to higher vigilance of patients in the current situation or a lack of resources.

Another limitation concerns the randomized group assignment, in which only a partly randomized allocation could be achieved. In addition, there was a comparably high dropout rate in IG2 that was potentially selective, which is typical for asynchronous web-based interventions [65,67,68]. It is possible that mothers with high self-efficacy and communication competency dropped out because they felt that they could not learn anything new. On the other hand, women with communication difficulties might have dropped out because they felt overwhelmed. Thus, the dropout might have caused an overestimation or underestimation of the effects [69]. In future studies, adequate measures should be taken to avoid dropouts. To summarize, both of the abovementioned limitations impaired the comparability of the 3 study groups. This should be considered when interpreting results and designing future studies.

### Conclusions

The evaluated digital interventions had different effects on communication behavior and patient safety. The intervention that was developed and delivered as a web-based training application appeared to be not sufficient in changing communication behavior in pregnant women and perceived patient safety risks when compared with a passive CG. Hence, it seems reasonable to combine the web-based application with other face-to-face interventions to achieve better effectiveness. Changes and adaptations to the existing web-based applications should be examined more closely in the future. In addition, more precise analyses of communication behavior and the interrelation of social cognitive determinants are warranted. Future research should control for more potential confounding variables, such as socioeducational status and prior knowledge of pregnancy and profession. Qualitative methods can be applied to gain more precise insights into the existing web-based applications to adjust. Future web application developers and researchers should also consider the mode of delivery and create a “native app” to make the intervention more accessible.

## Appendix

Multimedia Appendix 1 Comparison of the effectiveness of a web-based application with a digital live seminar to improve safe communication for pregnant women: 3-group partially randomized controlled trial.

Multimedia Appendix 2 CONSORT-eHEALTH checklist (V 1.6.1).

## Abbreviations

BCT: behavior change technique
CG: control group
G-BA: Gemeinsamer Bundesausschuss
HAPA: Health Action Process Approach
HCW: health care worker
IG1: live seminar group
IG2: web-based application group
T1: first time point
T2: second time point

## Data Availability

The data that support the findings of this study are available on request from the corresponding author, SL. The data were not allowed to be made publicly available because of privacy and data security reasons of the research participants.

## References

[1] Ritterband LM, Andersson G, Christensen HM, Carlbring P, Cuijpers P (2006). Directions for the International Society for Research on Internet Interventions (ISRII). J Med Internet Res.

[2] De Meester K, Verspuy M, Monsieurs KG, Van Bogaert P (2013). SBAR improves nurse-physician communication and reduces unexpected death: a pre and post intervention study. Resuscitation.

[3] Curtis LM, Mullen RJ, Russell A, Fata A, Bailey SC, Makoul G, Wolf MS (2016). An efficacy trial of an electronic health record-based strategy to inform patients on safe medication use: the role of written and spoken communication. Patient Educ Couns.

[4] Raynor DK, Blenkinsopp A, Knapp P, Grime J, Nicolson DJ, Pollock K, Dorer G, Gilbody S, Dickinson D, Maule AJ, Spoor P (2007). A systematic review of quantitative and qualitative research on the role and effectiveness of written information available to patients about individual medicines. Health Technol Assess.

[5] Wu H, Sun W, Huang X, Yu S, Wang H, Bi X, Sheng J, Chen S, Akinwunmi B, Zhang CJ, Ming WK (2020). Online antenatal care during the COVID-19 pandemic: opportunities and challenges. J Med Internet Res.

[6] Swindle T, Poosala AB, Zeng N, Børsheim E, Andres A, Bellows LL (2022). Digital intervention strategies for increasing physical activity among preschoolers: systematic review. J Med Internet Res.

[7] Calvert A, Vandrevala T, Parsons R, Barber V, Book A, Book G, Carrington D, Greening V, Griffiths P, Hake D, Khalil A, Luck S, Montague A, Star C, Ster IC, Wood S, Heath PT, Jones CE (2021). Changing knowledge, attitudes and behaviours towards cytomegalovirus in pregnancy through film-based antenatal education: a feasibility randomised controlled trial of a digital educational intervention. BMC Pregnancy Childbirth.

[8] van der Pijl MS, Kasperink M, Hollander MH, Verhoeven C, Kingma E, de Jonge A (2021). Client-care provider interaction during labour and birth as experienced by women: respect, communication, confidentiality and autonomy. PLoS One.

[9] Jolivet RR, Gausman J, Kapoor N, Langer A, Sharma J, Semrau KE (2021). Operationalizing respectful maternity care at the healthcare provider level: a systematic scoping review. Reprod Health.

[10] Attanasio LB, McPherson ME, Kozhimannil KB (2014). Positive childbirth experiences in U.S. hospitals: a mixed methods analysis. Matern Child Health J.

[11] Delaney AL, Singleton G (2020). Information and relationship functions of communication between pregnant women and their health care providers. Commun Stud.

[12] Kim SC, Boren D, Solem SL (2001). The Kim Alliance Scale: development and preliminary testing. Clin Nurs Res.

[13] Nicoloro-SantaBarbara J, Rosenthal L, Auerbach MV, Kocis C, Busso C, Lobel M (2017). Patient-provider communication, maternal anxiety, and self-care in pregnancy. Soc Sci Med.

[14] Rundell S (1991). A study of nurse-patient interaction in a high dependency unit. Intensive Care Nurs.

[15] Lippke S, Derksen C, Keller FM, Kötting L, Schmiedhofer M, Welp A (2021). Effectiveness of communication interventions in obstetrics-a systematic review. Int J Environ Res Public Health.

[16] Derksen C, Kötting L, Keller FM, Schmiedhofer M, Lippke S (2022). Psychological intervention to improve communication and patient safety in obstetrics: examination of the health action process approach. Front Psychol.

[17] Cheng ER, Carroll AE, Iverson RE, Declercq ER (2020). Communications between pregnant women and maternity care clinicians. JAMA Netw Open.

[18] Petersen Z, Nilsson M, Everett K, Emmelin M (2009). Possibilities for transparency and trust in the communication between midwives and pregnant women: the case of smoking. Midwifery.

[19] (2021). Global patient safety action plan 2021-2030. World Health Organization.

[20] Runciman W, Hibbert P, Thomson R, Van Der Schaaf T, Sherman H, Lewalle P (2009). Towards an international classification for patient safety: key concepts and terms. Int J Qual Health Care.

[21] Eulmesekian PG, Alvarez JP, Ceriani Cernadas JM, Pérez A, Berberis S, Kondratiuk Y (2020). The occurrence of adverse events is associated with increased morbidity and mortality in children admitted to a single pediatric intensive care unit. Eur J Pediatr.

[22] Matthias S (2018). APS-Weißbuch Patientensicherheit Sicherheit in der Gesundheitsversorgung: neu denken, gezielt verbessern.

[23] Shojania KG, Marang-van de Mheen PJ (2020). Identifying adverse events: reflections on an imperfect gold standard after 20 years of patient safety research. BMJ Qual Saf.

[24] Antony J, Zarin W, Pham B, Nincic V, Cardoso R, Ivory JD, Ghassemi M, Barber SL, Straus SE, Tricco AC (2018). Patient safety initiatives in obstetrics: a rapid review. BMJ Open.

[25] Gittell JH (2016). Relationships between service providers and their impact on customers. J Serv Res.

[26] Davis R, Campbell R, Hildon Z, Hobbs L, Michie S (2015). Theories of behaviour and behaviour change across the social and behavioural sciences: a scoping review. Health Psychol Rev.

[27] (2014). Every newborn: an action plan to end preventable deaths. World Health Organization.

[28] Schwarzer R (2008). Modeling health behavior change: how to predict and modify the adoption and maintenance of health behaviors. Appl Psychol.

[29] Schwarzer R, Luszczynska A (2020). Self-efficacy. Division of Cancer Control and Population Sciences.

[30] Kripalani S, LeFevre F, Phillips CO, Williams MV, Basaviah P, Baker DW (2007). Deficits in communication and information transfer between hospital-based and primary care physicians: implications for patient safety and continuity of care. JAMA.

[31] Lippke S, Wienert J, Keller FM, Derksen C, Welp A, Kötting L, Hofreuter-Gätgens K, Müller H, Louwen F, Weigand M, Ernst K, Kraft K, Reister F, Polasik A, Huener Nee Seemann B, Jennewein L, Scholz C, Hannawa A (2019). Communication and patient safety in gynecology and obstetrics - study protocol of an intervention study. BMC Health Serv Res.

[32] Derksen C, Dietl JE, Haeussler FE, Steinherr Zazo M, Schmiedhofer M, Lippke S (2022). Behavior change training for pregnant women's communication during birth: a randomized controlled trial. Appl Psychol Health Well Being (Forthcoming).

[33] Michie S, Richardson M, Johnston M, Abraham C, Francis J, Hardeman W, Eccles MP, Cane J, Wood CE (2013). The behavior change technique taxonomy (v1) of 93 hierarchically clustered techniques: building an international consensus for the reporting of behavior change interventions. Ann Behav Med.

[34] Gholami M, Schwarzer R, Reyes-Fernandez B, Rica C (2016). Brief scales for the multilingual assessment of HAPA variables. ResearchGate.

[35] Rider EA, Keefer CH (2006). Communication skills competencies: definitions and a teaching toolbox. Med Educ.

[36] Schwarzer R, Schuz B, Ziegelmann JP, Lippke S, Luszczynska A, Scholz U (2007). Adoption and maintenance of four health behaviors: theory-guided longitudinal studies on dental flossing, seat belt use, dietary behavior, and physical activity. Ann Behav Med.

[37] Keller FM, Derksen C, Kötting L, Schmiedhofer M, Lippke S (2021). Development of the perceptions of preventable adverse events assessment tool (PPAEAT): measurement properties and patients' mental health status. Int J Qual Health Care.

[38] Transforming different Likert scales to a common scale. IBM Corp.

[39] Gibson DG, Tamrat T, Mehl G (2018). The state of digital interventions for demand generation in low- and middle-income countries: considerations, emerging approaches, and research gaps. Glob Health Sci Pract.

[40] Walter B, Indreboe H, Lukasse M, Henriksen L, Garnweidner-Holme L (2021). Pregnant women's attitudes toward and experiences with a tablet intervention to promote safety behaviors in a randomized controlled trial: qualitative study. JMIR Form Res.

[41] Bogale B, Mørkrid K, Abbas E, Abu Ward I, Anaya F, Ghanem B, Hijaz T, Isbeih M, Issawi S, A S Nazzal Z, E Qaddomi S, Frøen JF (2021). The effect of a digital targeted client communication intervention on pregnant women's worries and satisfaction with antenatal care in Palestine-a cluster randomized controlled trial. PLoS One.

[42] Zhang CQ, Zhang R, Schwarzer R, Hagger MS (2019). A meta-analysis of the health action process approach. Health Psychol.

[43] Gourlan M, Bernard P, Bortolon C, Romain AJ, Lareyre O, Carayol M, Ninot G, Boiché J (2016). Efficacy of theory-based interventions to promote physical activity. A meta-analysis of randomised controlled trials. Health Psychol Rev.

[44] Schwarzer R (2016). Health Action Process Approach (HAPA) as a theoretical framework to understand behavior change. Actual Psicol.

[45] Tang MY, Smith DM, Mc Sharry J, Hann M, French DP (2019). Behavior change techniques associated with changes in postintervention and maintained changes in self-efficacy for physical activity: a systematic review with meta-analysis. Ann Behav Med.

[46] Lin H, Xu D, Yang M, Ma X, Yan N, Chen H, He S, Deng N (2022). Behaviour change techniques that constitute effective planning interventions to improve physical activity and diet behaviour for people with chronic conditions: a systematic review. BMJ Open.

[47] Hedin B, Katzeff C, Eriksson E, Pargman D (2019). A systematic review of digital behaviour change interventions for more sustainable food consumption. Sustain.

[48] Moshe I, Terhorst Y, Philippi P, Domhardt M, Cuijpers P, Cristea I, Pulkki-Råback L, Baumeister H, Sander LB (2021). Digital interventions for the treatment of depression: a meta-analytic review. Psychol Bull.

[49] Karamolahi PF, Bostani Khalesi Z, Niknami M (2021). Efficacy of mobile app-based training on health literacy among pregnant women: a randomized controlled trial study. Eur J Obstet Gynecol Reprod Biol X.

[50] Band R, Bradbury K, Morton K, May C, Michie S, Mair FS, Murray E, McManus RJ, Little P, Yardley L (2017). Intervention planning for a digital intervention for self-management of hypertension: a theory-, evidence- and person-based approach. Implement Sci.

[51] Mohr DC, Cuijpers P, Lehman K (2011). Supportive accountability: a model for providing human support to enhance adherence to eHealth interventions. J Med Internet Res.

[52] Sutton S (2008). How does the Health Action Process Approach (HAPA) bridge the intention–behavior gap? An examination of the model's causal structure. Applied Psychology.

[53] Yardley L, Choudhury T, Patrick K, Michie S (2016). Current issues and future directions for research into digital behavior change interventions. Am J Prev Med.

[54] Muuraiskangas S, Mattila E, Kyttälä P, Koreasalo M, Lappalainen R (2015). User experiences of a mobile mental well-being intervention among pregnant women. Proceedings of the 5th International Conference on Pervasive Computing Paradigms for Mental Health.

[55] Yardley L, Morrison L, Bradbury K, Muller I (2015). The person-based approach to intervention development: application to digital health-related behavior change interventions. J Med Internet Res.

[56] Kohl LF, Crutzen R, de Vries NK (2013). Online prevention aimed at lifestyle behaviors: a systematic review of reviews. J Med Internet Res.

[57] Chen Y, Tan D, Xu Y, Wang B, Li X, Cai X, Li M, Tang C, Wu Y, Shu W, Zhang G, Huang J, Zhang Y, Yan Y, Liang X, Yu S (2020). Effects of a HAPA-based multicomponent intervention to improve self-management precursors of older adults with tuberculosis: a community-based randomised controlled trial. Patient Educ Couns.

[58] Rollo S, Prapavessis H (2020). A combined health action process approach and mHealth intervention to increase non-sedentary behaviours in office-working adults-a randomised controlled trial. Appl Psychol Health Well Being.

[59] King D, Miller C (2022). P053 using the health action process approach theoretical framework to predict and explain dietary behaviors in a worksite diabetes prevention intervention. J Nutr Educ Behav.

[60] Laranjo L, Dunn AG, Tong HL, Kocaballi AB, Chen J, Bashir R, Surian D, Gallego B, Magrabi F, Lau AY, Coiera E (2018). Conversational agents in healthcare: a systematic review. J Am Med Inform Assoc.

[61] Montenegro JL, da Costa CA, da Rosa Righi R (2019). Survey of conversational agents in health. Expert Syst Appl.

[62] Hauser-Ulrich S, Künzli H, Meier-Peterhans D, Kowatsch T (2020). A smartphone-based health care chatbot to promote self-management of chronic pain (SELMA): pilot randomized controlled trial. JMIR Mhealth Uhealth.

[63] Duplaga M, Tubek A (2018). mHealth - areas of application and the effectiveness of interventions. Zdr Publiczne Zarz.

[64] Grady A, Yoong S, Sutherland R, Lee H, Nathan N, Wolfenden L (2018). Improving the public health impact of eHealth and mHealth interventions. Aust N Z J Public Health.

[65] Wu MS, Chen SY, Wickham RE, Leykin Y, Varra A, Chen C, Lungu A (2022). Predicting non-initiation of care and dropout in a blended care CBT intervention: impact of early digital engagement, sociodemographic, and clinical factors. Digit Health.

[66] Carroll JK, Moorhead A, Bond R, LeBlanc WG, Petrella RJ, Fiscella K (2017). Who uses mobile phone health apps and does use matter? A secondary data analytics approach. J Med Internet Res.

[67] Torous J, Lipschitz J, Ng M, Firth J (2020). Dropout rates in clinical trials of smartphone apps for depressive symptoms: a systematic review and meta-analysis. J Affect Disord.

[68] Bremer V, Chow PI, Funk B, Thorndike FP, Ritterband LM (2020). Developing a process for the analysis of user journeys and the prediction of dropout in digital health interventions: machine learning approach. J Med Internet Res.

[69] Eysenbach G (2005). The law of attrition. J Med Internet Res.

