# Supplementary material for: Comparing the Effectiveness of a Web-Based Application With a Digital Live Seminar to Improve Safe Communication for Pregnant Women: 3-Group Partially Randomized Controlled Trial
Source: JMIR Pediatr Parent. 2023 Jul 24;6:e44701. doi: 10.2196/44701 (PMC10407768; doi:10.2196/44701)
Supplement: Multimedia Appendix 1 [file pediatrics_v6i1e44701_app1.docx]

**Appendix S1**

Specifications of live seminar training provided to pregnant women and their partners [^32^].

|  | Details | Further information |
| --- | --- | --- |
| Purpose | Facilitate communication between those receiving and providing obstetrics care. |  |
| Target group | Pregnant women and their partners |  |
| Length | 2.5 hours |  |
| Active period | June 2020 to August 2021 |  |
| Modules | Preparation for session | Participants complete self-reflection questionnaire to identify needs and preferences of birthing. |
|  | Introduction round | Participants present “ideal” birth to group. |
|  | Perspective taking | Participants construct empathy maps for different stakeholders involved in birthing process. |
|  | Speaking Up | Participants are taught how to voice concerns in obstetrics setting. |
|  | Closed-Loop | Participants are taught how to effectively relay information to health care workers. |
|  | Action planning | Participants develop a plan to practice and apply tools and strategies covered. |

**S2**

Specifications of the TeamBaby web app

|  | Details | Further information |
| --- | --- | --- |
| Purpose | Facilitate communication between those receiving and providing obstetrics care. |  |
| Target group | Pregnant women and their partners |  |
| Length | 2.00 hours |  |
| Active period | February 2021 to June 2022 |  |
| Modules | Preparation for lessons | Participants complete self-reflection questionnaire to identify needs and preferences of birthing. |
| Lesson 1 | Communication competencies | Introduction and explanation of safe communication (behaviour) with multiple choice. |
| Lesson 2 | 4 sides of communication | This exercise is about reducing the chances of a misunderstanding with the communication partner. Situation examples are provided. Answer format: open questions and multiple-choice answers. |
| Lesson 3 | Speaking Up | Practical lesson with the goal of expressing own wishes, as soon as their needs are at risk. Answer format: Multiple choice and open questions with typical situations during the birth process. |
| Lesson 4 | Closed-Loop | Practical lesson - how to avoid misunderstandings with the “close-the-loop” strategy.  The main goal is, how to ask specific questions when something is unclear. Answer format: multiple choice with an example communication process between doctor and mother to be as well as self-reflection questions. |
| Lesson 5 | Empathy and change of perspective | Lesson about the emotional side of communication. Empathy is crucial to being well supported during childbirth. Putting yourself in the shoes of midwives, doctors and support persons in terms of feelings, fears, concerns and tasks. Answer format: open questions. |
| Lesson 6 | Communication strategy ISBAR | Lesson with an exercise how to communicate concerns quickly and effectively. One way to do this is to learn how to effectively structure content. Answer format: open questions and multiple choice guided by an example situation. |
| Lesson 7 | 5-point preparation | Lesson concerning best preparation for conversations and appointments with medical professionals. The goal is a good preparation for an effective doctor-patient relationship. Answer format: open questions considering the presented 5-point preparation. |
| Lesson 8 | Active stress management | Lesson - how to deal with stressful situations (even after giving birth) and what tricks of the trade can be used. Answer format: multiple choice considering an example situation as well as example dialogue between expectant mother and support person. |
| Lesson 9 | (Behavioural) action planning | Lesson in which participants develop a plan to practice and apply tools and strategies to communicate safely. |
| Lesson 10 | Repetition | Repetition of Lesson 1-9 with multiple choice as well as rating questions concerning the own competencies. |

**S3**.

Intercorrelation of safe communication behavior and social cognitive Health Action Process Approach variables controlled for age, marital status, education level, and nationality per group.

| Mode | | | 1 | 2 | 3 | 4 | 5 | 6 | 7 | 8 | 9 |
| --- | --- | --- | --- | --- | --- | --- | --- | --- | --- | --- | --- |
| **Perceived patient safety at T1** | | | | | | | | | | | |
|  | | **IG1^a^** | | | | | | | | | |
|  |  | *r* | 1 | ^g^— | — | — | — | — | — | — | — |
|  |  | *P* value | — | — | — | — | — | — | — | — | — |
|  | | **CG^b^** | | | | | | | | | |
|  |  | *r* | 1 | — | — | — | — | — | — | — | — |
|  |  | *P* value | — | — | — | — | — | — | — | — | — |
|  | | **IG2^c^** | | | | | | | | | |
|  |  | *r* | 1 | — | — | — | — | — | — | — | — |
|  |  | *P* value | — | — | — | — | — | — | — | — | — |
| **Perceived patient safety at T2** | | | | | | | | | | | |
|  | | **IG1** | | | | | | | | | |
|  |  | *r* | 0.310^d^ | 1 | — | — | — | — | — | — | — |
|  |  | *P* value | <.001 | — | — | — | — | — | — | — | — |
|  | | **CG** | | | | | | | | | |
|  |  | *r* | 0.203 | — | — | — | — | — | — | — | — |
|  |  | *P* value | .03^e^ | — | — | — | — | — | — | — | — |
|  | | **IG2** | | | | | | | | | |
|  |  | *r* | 0.238 | — | — | — | — | — | — | — | — |
|  |  | *P* value | .054 | — | — | — | — | — | — | — | — |
| **Communication behavior at T1** | | | | | | | | | | | |
|  | | **IG1** | | | | | | | | | |
|  |  | *r* | 0.146 | 0.062 | 1 | — | — | — | — | — | — |
|  |  | *P* value | .12 | .509 | — | — | — | — | — | — | — |
|  | | **CG** | | | | | | | | | |
|  |  | *r* | 0.094 | 0.065 | — | — | — | — | — | — | — |
|  |  | *P* value | .33 | .507 | — | — | — | — | — | — | — |
|  | | **IG2** | | | | | | | | | |
|  |  | *r* | −0.063 | 0.167 | — | — | — | — | — | — | — |
|  |  | *P* value | .62 | .181 | — | — | — | — | — | — | — |
| **Communication behavior at T2** | | | | | | | | | | | |
|  | | **IG1** | | | | | | | | | |
|  |  | *r* | 0.055 | −0.040 | 0.389 | 1 | — | — | — | — | — |
|  |  | *P* value | .56 | .669 | <.001^d^ | — | — | — | — | — | — |
|  | | **CG** | | | | | | | | | |
|  |  | *r* | −0.075 | −0.245 | 0.382 | — | — | — | — | — | — |
|  |  | *P* value | .44 | .011^e^ | <.001^d^ | — | — | — | — | — | — |
|  | | **IG2** | | | | | | | | | |
|  |  | *r* | −0.701 | −0.193 | 0.415 | — | — | — | — | — | — |
|  |  | *P* value | .57 | .121 | <.001^d^ | — | — | — | — | — | — |
| **Coping self-efficacy at T1** | | | | | | | | | | | |
|  | | **IG1** | | | | | | | | | |
|  |  | *r* | 0.008 | −0.034 | 0.091 | 0.099 | 1 | — | — | — | — |
|  |  | *P* value | .93 | .719 | .338 | .297 | — | — | — | — | — |
|  | | **CG** | | | | | | | | | |
|  |  | *r* | −0.200 | −0.118 | 0.309 | 0.328 | — | — | — | — | — |
|  |  | *P* value | .04^e^ | .224 | .001^d^ | <.001^d^ | — | — | — | — | — |
|  | | **IG2** | | | | | | | | | |
|  |  | *r* | −0.154 | 0.103 | 0.408 | 0.346 | — | — | — | — | — |
|  |  | *P* value | .22 | .409 | <.001^d^ | .004^f^ | — | — | — | — | — |
| **Coping self-efficacy at T2** | | | | | | | | | | | |
|  | | **IG1** | | | | | | | | | |
|  |  | *r* | −0.034 | −0.042 | 0.094 | 0.464 | 0.180 | 1 | — | — | — |
|  |  | *P* value | .72 | .654 | .321 | <.001^d^ | .055 | — | — | — | — |
|  | | **CG** | | | | | | | | | |
|  |  | *r* | −0.195 | −0.376 | 0.092 | 0.441 | 0.368 | — | — | — | — |
|  |  | *P* value | .05^e^ | <.001^d^ | .341 | <.001^d^ | .001^d^ | — | — | — | — |
|  | | **IG2** | | | | | | | | | |
|  |  | *r* | −0.097 | −0.068 | 0.265 | 0.576 | 0.350 | — | — | — | — |
|  |  | *P* value | .44 | .586 | .032^e^ | <.001^d^ | .004^f^ | — | — | — | — |
| **Intention at T1** | | | | | | | | | | | |
|  | | **IG1** | | | | | | | | | |
|  |  | *r* | −0.003 | 0.035 | 0.391 | 0.102 | 0.021^e^ | −0.084 | 1 | — | — |
|  |  | *P* value | .97 | .710 | <.001^d^ | .279 | .897 | .375 | — | — | — |
|  | | **CG** | | | | | | | | | |
|  |  | *r* | −0.157 | 0.054 | 0.240 | 0.264 | 0.424 | 0.064 | — | — | — |
|  |  | *P* value | .10 | .581 | .012^e^ | .006^f^ | <.001^d^ | .509 | — | — | — |
|  | | **IG2** | | | | | | | | | |
|  |  | *r* | −0.072 | 0.048 | 0.431 | 0.338 | 0.546 | 0.166 | — | — | — |
|  |  | *P* value | .57 | .705 | <.001^d^ | .006^f^ | <.001^d^ | .184 | — | — | — |
| **Coping planning at T1** | | | | | | | | | | | |
|  | | **IG1** | | | | | | | | | |
|  |  | *r* | 0.140 | −0.027 | 0.288 | 0.177 | 0.125 | 0.010 | 0.091 | 1 | — |
|  |  | *P* value | .27 | .776 | .002^f^ | .060 | .187 | .918 | .335 | — | — |
|  | | **CG** | | | | | | | | | |
|  |  | *r* | −0.128 | 0.045 | 0.340 | 0.311 | 0.410 | 0.157 | 0.320 | — | — |
|  |  | *P* value | .19 | .645 | <.001^d^ | .001^d^ | <.001^d^ | .105 | <.001^d^ | — | — |
|  | | **IG2** | | | | | | | | | |
|  |  | *r* | −0.206 | 0.107 | 0.634 | 0.374 | 0.490 | 0.252 | 0.570 | — | — |
|  |  | *P* value | .10 | .394 | <.001^d^ | .002^f^ | <.001^d^ | .041^e^ | <.001^d^ | — | — |
| **Coping planning at T2** | | | | | | | | | | | |
|  | | **IG1** | | | | | | | | | |
|  |  | *r* | −0.075 | −0.276 | 0.187 | 0.456 | 0.038^e^ | 0.581 | 0.034 | −0.012 | 1 |
|  |  | *P* value | .43 | .003^f^ | .046^e^ | <.001^d^ | .688 | <.001^d^ | .721 | .898 | — |
|  | | **CG** | | | | | | | | | |
|  |  | *r* | −0.150 | −0.298 | 0.110 | 0.578 | 0.207 | 0.683 | 0.162 | 0.151 | — |
|  |  | *P* value | .12 | .002 | .256 | <.001^d^ | .032^e^ | <.001^d^ | .094 | .118 | — |
|  | | **IG2** | | | | | | | | | |
|  |  | *r* | −0.144 | −0.195 | 0.225 | 0.543 | 0.329 | 0.784 | 0.155 | 0.322 | — |
|  |  | *P* value | .25 | .117 | .069 | <.001^d^ | .007^f^ | <.001^d^ | .214 | .008^f^ | — |

^a^IG1: live seminar group.

^b^CG: control group.

^c^IG2: web-based application group.

^d^Correlation is significant at the .001 level.

^e^Correlation is significant at the .05 level.

^f^Correlation is significant at the .01 level.

^g^Not applicable

**S4**

# **Table** 4: Differences in socio-demographic variables between groups

|  | Sociodemographic data | ^a^χ² | *^b^df* | *^c^p* |
| --- | --- | --- | --- | --- |
|  | Nationality | 5.486 | 4 | .241 |
|  | Age | 4.165 | 4 | .384 |
|  | Marital status | 4.479 | 6 | .612 |
|  | Education | 8.311 | 12 | .760 |

^a^Chi-square statistics

^b^degrees of freedom

^c^p-value

**S5**

**Table 5**. Comparison of participants who dropped out and participants who provided T2 data.

| Intervention group | Sociodemographic data | ^a^χ² | ^b^df | ^c^p |
| --- | --- | --- | --- | --- |
|  | Nationality | 0.188 | 1 | .665 |
|  | Age | 2.990 | 3 | .393 |
|  | Marital status | 1.432 | 2 | .489 |
|  | Education | 5.381 | 6 | .496 |
| Control group | Sociodemographic data | χ² | df | p |
|  | Nationality | 2.161 | 2 | .339 |
|  | Age | 3.533 | 2 | .171 |
|  | Marital status | 1.502 | 3 | .682 |
|  | Education | 7.891 | 5 | .162 |
| App group |  |  |  |  |
|  | Nationality | 5.936 | 1 | ^d^.015 |
|  | Age | 49.693 | 3 | ^e^<.001 |
|  | Marital status | 2.282 | 2 | .320 |
|  | Education | 3.244 | 5 | .662 |

^a^Chi-square

^b^degrees of freedom

^c^p-value

^d^significant at P = .05 level

^e^significant at P = .001 level
